# Medical Students’ Perceptions towards Digitization and Artificial Intelligence: A Mixed-Methods Study

**DOI:** 10.3390/healthcare10040723

**Published:** 2022-04-13

**Authors:** Adrian Gillissen, Tonja Kochanek, Michaela Zupanic, Jan Ehlers

**Affiliations:** 1 Institute for Didactics and Educational Research in Health Care, Department of Medicine, Faculty of Health, Witten/Herdecke University, 58455 Witten, Germany; tonja.kochan3k@gmail.de (T.K.); jan.ehlers@uni-wh.de (J.E.); 2 Interprofessional and Collaborative Didactics, Department of Medicine, Faculty of Health, Witten/Herdecke University, 58455 Witten, Germany; michaela.zupanic@uni-wh.de

**Keywords:** medical students, perceptions, digitization in medicine, artificial intelligence

## Abstract

Digital technologies in health care, including artificial intelligence (AI) and robotics, constantly increase. The aim of this study was to explore attitudes of 2020 medical students’ generation towards various aspects of eHealth technologies with the focus on AI using an exploratory sequential mixed-method analysis. Data from semi-structured interviews with 28 students from five medical faculties were used to construct an online survey send to about 80,000 medical students in Germany. Most students expressed positive attitudes towards digital applications in medicine. Students with a problem-based curriculum (PBC) in contrast to those with a science-based curriculum (SBC) and male undergraduate students think that AI solutions result in better diagnosis than those from physicians (*p* < 0.001). Male undergraduate students had the most positive view of AI (*p* < 0.002). Around 38% of the students felt ill-prepared and could not answer AI-related questions because digitization in medicine and AI are not a formal part of the medical curriculum. AI rating regarding the usefulness in diagnostics differed significantly between groups. Higher emphasis in medical curriculum of digital solutions in patient care is postulated.

## 1. Introduction

Digitized health care systems require all players to acquire suitable knowledge of how to use these technologies appropriately and to understand their implications on patient management in general as well as on a case-by-case basis. Medical knowledge is expanding exponentially and requires physicians to be constantly up-to-date and quickly communicate, analyze, and recall medical information from numerous sources. Since 1955, artificial intelligence (AI) has had more and more support from stakeholders in the medical field and elsewhere to generate and investigate digital data at a speed and precision never seen before [1]. Digitization, including AI, changes not only the physician’s work but requires also that medical education must align with these quite different health care contexts compared to traditional teaching concepts [2]. Further, non-analytical, humanistic aspects of medicine come under scrutiny and compete with digital technologies. The acceptance of advanced technologies by students and health professionals and the weighing of its usefulness is extremely important once this modality of healthcare delivery became an integral part of mainstream healthcare. Acceptance, keenness to use the digital tools, knowledge and skills, as well as an exuberance to utilize digital tools as an inherent way of service delivery by healthcare professionals, particularly by doctors, help facilitate the integration of eHealth and thus enhance the quality of health care [3]. This means that among other institutions, such as universities, more and more should ensure that all active players, including medical students, acquire knowledge, skills, and attributes to work with these digital tools by an adaptation of curricula of education [4,5,6,7]. Although scientific publication on AI has increased since the beginning of this century, integration into medical curriculum for better understanding of AI algorithms and how to maximize their use is rudimentary [8].

Studies have demonstrated the usefulness of AI algorithms in various medical specialties, including radiology, ophthalmology, dermatology, pathology and pulmonary medicine [9,10]. Regardless of the paucity of evidence to support digital tools, including AI, in day-to-day routine in patient care and irrespective of the likeliness of the rapid emergence of numerous AI applications, students’ contact with university and medical courses teaching these concepts are rare. Surveys investigating students’ attitudes towards field-specific AI are just emerging [9,11,12,13,14]. In some studies, students indicate their intention to abstain from medical fields, such as radiology, where AI was regarded as a potential competitor to physicians’ work [9,11]. However, most wish for the integration of digital applications and smart algorithms as well as their use in clinical practice and integration into their curriculum [2,8,9,15,16].

No study has tested—to the best of the authors’ knowledge—whether students’ perceptions regarding various aspects of eHealth (digitization including AI) depends on only their personal beliefs or also on other confounding factors. Lee et al. (2021) found there is little consensus on what and how to teach AI in medical education, requiring further research to facilitate greater implementation of standardized aspects of digital medicine and AI in the medical curriculum [17], while German universities offer medical studies either a science-based focus (SBC, science-based curriculum) or a problem-based curriculum (PBC), which gives the unique opportunity to evaluate students’ perceptions in this regard, allowing the analysis of compounding factors not only regarding gender and training stage but also the curriculum type.

## 2. Aim of the Study

The overall objective of this study was to investigate today’s medical students’ attitudes towards AI and other digital working tools. We wanted to understand if age, gender, semester level, and curriculum type influence their views. This study also assembled information on students’ understanding of AI algorithms and digital applications in health care and assessed their level of confidence in working alongside these tools after graduation into patient care. It is our belief that this information may possess the means to employ digital tools, including AI, into the curriculum of medical students efficiently, enhancing their confidence in using them and therefore better equipping our future physicians with sufficient knowledge.

## 3. Materials and Methods

### 3.1. Design

In order to best pursue the aim of this study, an exploratory mixed-method design was used [18,19]. We used a sequential exploratory strategy in which a qualitative study phase was followed by a quantitative survey [20,21,22]. The intention of the initial qualitative component of the first study phase was to collect information about medical students’ perceptions regarding digitization and artificial intelligence (AI) in medicine. This was then integrated into the second study phase consisting of a nationally representative sample of the same sort of cohort. Thus, the first phase informed the next in an additive form, but it is not a parallel design per se. This design is widely used to evaluate the effect of community influence in which one method enriches the other method for comprehensibility [23]. For the first phase, themes were extracted from the literature related to medical students’ perceptions regarding digitization in medicine, eHealth, and AI. The following topics were extracted:

- Patient-related themes: digitization in patient self-management and interaction with the health-care system;
- Physician-related themes: communication, information, managing health data, AI and machine learning, and patient and administrative management;
- Student-related themes: course of digitization and AI in medical school and attitudes towards the digitization of medicine.

This information was then analyzed in two discussion group sessions among all authors, and a resulting interview guide with a set of open questions about medical students’ perceptions of digitization and artificial intelligence was constructed. This set consisted of three main themes, and the authors agreed on adjunct questions for each theme to probe explanations of the answers more deeply. The list was piloted with five medical students, allowing further refinement prior to the interviews. The items are listed in Table S1.

For the second, quantitative study phase, this findings were used to develop an internet-based survey to confirm the results of the qualitative part quantitatively but not to generate a formal theory [21,24]. Every item that was mentioned in more than two interviews was translated into a question. All questions were reviewed by the authors separately for content validity. This is seen as an objective judgment about the construct of an instrument, ensuring the instrument’s relevance to the study’s aim and elucidating how to express phrases, the wording of questions, and understanding the researcher’s intended concept [25,26]. The items were then tested through a pilot study consisting of a group of 4 pre- and 4 clinical students, mediated by the authors to understand how they perceive the subject of interest and in order to finalize the list of items. The comments and suggestions were integrated, and overlaps were avoided, resulting in the final construct of questions.

### 3.2. Participants and Selection Criteria in Each Phase

In Germany, digitization and AI are not a formal part of the medical curriculum although some medical students may have acquired relevant information about these themes during courses with patient presentation (hidden curriculum). All in all, medical students were, in terms of the curriculum, digitally naive. All participants of the first phase were students from their 1st to 6th year (undergraduate, 1st to 2nd year; graduate, 3rd to 6th year) from German universities. The inclusion criteria were their active study of medicine and their agreement for their voluntarily participation. In the same way, the exclusion criteria were suspension their studies as well as other exceptional situations. Prior to start, informed consent was obtained, which was followed by the collection of telephone numbers and email addresses. Convenience sampling was used. They were selected purposely and consecutively, in part by snowball until theoretical saturation was reached. All were approached personally by the authors. Once started, no interviewee dropped out of the interview, which lasted about 30 min. Semester number and interview time were comparable between the two groups. Table 1 summarizes the baseline characteristics of all participants. All quotations in this paper are translations from German language into English.

For the second, quantitative study phase, identical inclusion/exclusion criteria applied. The online survey was sent to all medical faculties in Germany, from which most forwarded the survey invitation by email to about 80,000 medical students to fulfill the principle of maximum diversity through convenience sampling method. Each contained an invitation letter and an information sheet. To avoid a potentially low response rate, 280 Amazon vouchers, each for EUR 25 per completed survey, were offered as incentives, which were distributed by way of a lottery. The samples of qualitative and quantitative studies are comparable in age and percent number of PBC/SBC students but slightly different in gender distribution and frequency of undergraduate or graduate semester (Table 1).

### 3.3. Analytical Strategy of the Qualitative Phase

The interviews consisted of semi-structured face-to-face or telephone interviews. They took place between November 2019 and March 2020 at the Witten/Herdecke University. Students replies were transcribed as verbatim texts and analyzed using an inductive coding approach according to Mayring’s principles, as also exploited by others [27,28,29], aided by the use of Quirkos 2.4 software (Quirkos, Edinburgh, United Kingdom www.quirkos.com accessed on 11 August 2021). A thematic analysis was performed by all authors and themes linked and grouped to develop a schema for interpreting the data, ensuring rigor in analysis [30]. When the perceptive content of the interviewees replicated itself, data saturation was assumed, and the interview series was terminated. A.G. and J.E. read each transcript up to three times to familiarize themselves with the contents and in order to analyze the content properly. Data were then independently coded (Table 2). The process involved the recognition of patterns and connections across the data and the establishment of themes and sub themes that were pertinent and applicable to the whole data set. Differences were discussed under the facilitation of TK until general consensus was achieved. Reflexivity was maintained by the three researchers involved in the data analysis, being cognizant throughout of their own personal context as, respectively, practicing clinicians and educators and of any potential effect this may have had on their interpretation of the data. Using this methodological approach, the authors followed a quantitative inquiry approach, which is also the cornerstone of grounded theory [31].

### 3.4. Analytical Strategy of the Quantitative Phase

The questionnaire consists of a total of 71 questions in eight sections: (A) sociodemographics, (B) preliminary activity, (C) admission to medical studies, (D) medical studies, (E) expectations of studies/profession, (F) learning, (G) future and digitization, and (H) patient and error management. Likert scale questions (ranging from 0 = decline/do not know to 7 = completely agree), questions with a percent scale from 0–100, and questions with the option of three answers (do not know, false, fully agree) were used. An item was considered a “firm perception” when the mean response was within one-third of the lowest/highest possible answer scores. The survey took place between September 2020 and January 2021.

### 3.5. Statistics

Statistical analysis was performed in the quantitative study part using SPSS (V27). Descriptive statistics are presented in percentages. An unpaired, two-tailed Wilcoxon rank-sum test was carried out to compare the responses relating to perceptions in digitization in medicine and AI. Group comparators were curriculum type (PBC vs. SBC), gender (female vs. male), and semester levels. A *p*-value of less than 0.05 was considered statistically significant.

## 4. Results

Students estimated that digital health cannot and will never replace traditional health services and medical consultations in total, but it will change the way doctors and patients will deal with each other.

“*I think, in the digital age the personal contact is particularly important. Many [patients] can easily search for information in the Internet using their mobile or smartphone. But it is something different when patients and doctors interact with each other and communicate in person. The physician can do a physical examination, take care of the patient directly which allows also emphatic interaction into the patient’s psyche*”.

This perception is mirrored by the data from the quantitative study. Digitization in general is not seen as a competition for doctors but as an accessory tool to improve their performance, save time, and make their work easier. Male students are somewhat more skeptical than women (Table 3). Male students see AI more as an encumbrance than as useful assistance.

Although the semi-structured interview was based only on three major topics, students discussed six related sub-themes in lengths and with great enthusiasm, which were categorized as the digital patient, digitization in doctor–patient interaction, technical aspects of digitization, robotics in medicine, artificial intelligence (AI), and digitization in university.

### 4.1. The Digital Patient

Students show a well-balanced attitude or are even enthusiastic regarding the advantages of internet-using patients (or “ePatients”, as Masters, 2017, put it [32]). Concerns are related to potentially unreliable and non-certified internet sources eventually causing confusion in the patient–doctor relationship, particularly when the doctor disagrees with the patient’s internet inquiry (Figure S1). In general, they believe that informed patients can more easily be integrated into the doctor’s decision making.

“*That means that the patient visits the doctor well informed. Informed patients gave thoughts to their symptoms, in a positive but also possibly in a negative sense. As a matter of principle I like informed patients as long as patients are open for further suggestions and towards the doctor’s medical advice. On the flip side can such lay information interfere with doctor’s intention because it cause a behavioral bias on the patient side towards certain diagnostic procedures and therapies*”.

Some doubt the reliability of health apps and the practical usefulness for doctors, particularly those who lack the necessary willingness and technical understanding.

“*But I must say, for example just for me, I am not very technically avid and only partially trained or have only meager digital skills*”.

Students emphasize that apps might be used as a useful information source for doctors as well as for patients although they question the accuracy of mobile health applications for patients [33], and they caution a possible fallout for the utilization on the health care system (Table S2). Only a minority of the students knew that common activity trackers are not certified medical devices, precluding them from being used as such. Students argue in favor of the use of those devices, mainly citing the stimulating effect on a physical activity and their perception of these devices as a positive motivation tool for healthy lifestyle (Table S3).

Although the quantitative study part did not find ample differences, SBC students tend to have slightly more restrictive attitude than PBC students towards patients’ use of consumer health apps. They seem, although by and large having a more positive than a timid attitude, more reluctant regarding the use of medical apps to aid diagnosis and therapy by doctors. Interestingly, the perception for or against the use of digital applications in medicine for patients and doctors seems to change. While undergraduate students have more critical and restrained perceptions, graduate students see more of the positive side most likely due to their comparably higher training level. Thus, group differences of perceptions were mainly driven by semester rank rather than by gender or by educational type (Table 3).

### 4.2. Digitization in Doctor–Patient Interaction

In interviews, participants verbalized indifferent knowledge of telemedicine inventions. Positive aspects included the simplification of doctor consultations, particularly in sparsely populated areas, possible 24/7 doctor access, and the medical on-the-spot support of paramedics (Table S3, supplement). They doubted that electronic communication services would enhance the doctor–patient relationship because direct and physical doctor–patient interaction will always be the cornerstone of patient care. However, increasing electronic communications, in contrast to face-to-face contact between patients and the doctor but also between stakeholders in medicine, is seen as unavoidable in modern days. The computer screen might be on the verge of becoming more essential than the physical presences of the patient, or personal interactions might weaken, such as the deterioration of experience of physical examination and medical history taking due to the dominance of electronic data and the loss of individual patient characteristics.

**Figure 1:**
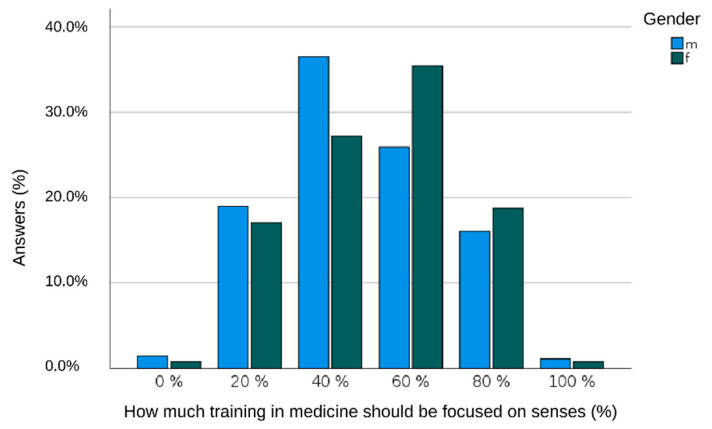
Male (m) students favor high-tech medical themes in the curriculum more than women do (f).

Students of the quantitative study part had positive attitudes toward telemedicine, with women having the most favorable views. Digital communication and attentiveness toward patients despite working with a computer and electronic networking were seen neither overly optimistically nor pessimistically within all groups (Table 4). Interestingly, all of them think that digital solutions in patient care might ease doctor–nurse communications but not personal doctor–patient interactions. Male students favor high-tech medicine themes in the curriculum, while female students prefer the personal patient–doctor-interaction and use their senses in physical examinations rather than relying on impersonal technical tools for the diagnostic workup (Figure 1). All students, and particularly those at the graduate level, express their willingness to improve healthcare, including its digital solution concepts.

### 4.3. Technical Aspects of Digitization

Students had a balanced attitude towards technical and operational aspects of digitization in patient care, citing critical but also positive aspects as summarized in Table S5. All students were aware of privacy issues and considered informed consent as essential. However, they do not appear to give privacy issues a high ranking order because both groups cite that many people easily give away personal information voluntarily, such as in social media or while using open Internet access gates (Table S6). They even consider privacy regulations as somewhat cumbersome for the doctors to whom patients have to give private information anyway.

“*I don’t know why data protection concerns in medicine are so widely discussed. Well, I don’t care if my health insurance and physicians can see my diseases because they get this information anyway. I you ask me … I tell my physician my medical problems anyway which is a courtesy making life easier*”.

Most students reject the notion that digitization interferes substantially with privacy. They further regard data storage on personal health insurance cards as more helpful for doctors’ work and as offering less vulnerability for abuses. They see a high potential for enhancing medical quality, which out-weighs the risks. Students in earlier semesters view cybersecurity and women in particular view insurance cards with more concern than do graduate students or men, respectively (Table 4).

### 4.4. Robotics and AI in Medicine

The interviewees did not clearly distinguish robotics and machine learning from other AI applications. Most students draw their knowledge about AI and medical robotic systems either from personal experience or interest by citing the movie *I, Robot*, from casual encounters during courses, or from reports in the general media. Particularly, SPC students expressed critical attitudes against AI and robotic applications in medicine, which they regard as inhuman. In contrast, PBC students saw AI and robotics as supportive, even expressing excitement. Both groups strongly emphasized that AI and robots will never replace doctors and the warm heartedness of human-to-human interaction although AI might be a formidable competitor particularly in radiology, pathology, and other fields were AI has been shown to outperform even specialists (Table S7).

“*I think, an intelligent android will never replace a physician because the human element is always the most important component in a doctor-doctor-interaction. Solid social contacts, empathy but also tactfulness is so important which can never be accomplished by a robot*”.

“*I think, that’s difficult, because I feel a certain emotional suspiciousness towards technical solutions and AI. But objectively and pragmatically seen, these digital assistance solutions are as a matter of principle a good thing. But emotionally I am quite wary*”.

“*Yes, artificial intelligence is a very fascinating area of cutting edge new technological developments. I think, the we can profit enormously from AI*”.

Depending on the analyzed group, 10% to almost 40% of students felt uninformed about AI and therefore could not answer the questions of the quantitative survey (Table 5A–C). More PBC than SBC students and more graduate than undergraduate students (non-significant after Bonverroni correction) believe that physicians will lose their medical skills through AI applications. Around 70% of students think that to some extent, AI generates better diagnoses in rare diseases. This study found significant differences between groups: SBC students, women, and undergraduate students are less convinced that AI is superior to physicians (comparison between all groups *p* < 0.001, Table 5B). In contrast to the qualitative part, PBC and SBC students were equally uneasy to disapprove AI (Table 5C). Men as well as undergraduate students had a significant more pessimistic perception towards AI than their counterparts (*p* = 0.002 and *p* < 0.002, respectively).

## 5. Discussion

This study, based on an exploratory sequential analysis consisting of two study parts, investigated students’ attitudes towards various aspects of digitization in medicine with the focus of AI. Germany and many other countries, there is a lack of AI and other digital solutions for patient care in the curriculum, which provides only a cursory reference to AI at the most despite its advantages and its more frequent use [32,34]. Therefore, this study adds to our understanding of what medical students think about chances and challenges of digital tools in patient management as well as the role and future of AI in medicine. Without a structured curriculum, it is difficult to select a solid knowledge base on these themes, which easily can explain the helplessness of some students of our online survey. However, medicine still has to deal with the adoption of digital working tools, including the integration of high-tech simulation into medical curriculum [2,35,36].

The students of the qualitative phase revealed that they drew their knowledge and attitude regarding AI and robotics/machine learning, which they could not clearly differentiate from media and films, and not from courses in the university. Regardless, the qualitative interviews revealed a great array of detailed opinions ranging from AI as a potential competitor in certain medical fields such as radiology, pathology, and others to being supportive for physician’s work, liability, and data security. The quantitative study part further revealed for the first time, to the authors knowledge, that students’ attitudes are not to be contemplated from a standpoint of structural unity but that distinctive stances exist. Significantly more PBC than SBC and more graduate than undergraduate students think that AI will hamper medical skills of physicians, and significantly more PBC students, male students, and graduate students are convinced of the superiority of AI in detecting rare diseases. Although up 38% of our students could not answer the AI questions in the quantitative survey—initially brought up in the interviews of the qualitative study part—it seems encouraging in comparison to an earlier survey, which reported that about 70% of respondents were unaware of AI topics in medicine [16]. Students of this study expressed a great interest in integrate digitization, AI, and machine learning into the medical curriculum, which is in concordance with earlier reports [9,14,15,16]. At least the qualitative part of this study matches nicely, from the students’ perspective, the attitudes from faculty members in German medical faculties because both postulate an intensification of AI competence in medical training [37].

Healthcare is currently undergoing a digital transformation. Therefore, it is imperative to leverage digital technologies to further improve our understanding of disease pathogenesis, diagnosis, and therapy. Stakeholders in medicine need to believe that new technologies provide an advantage to traditional working structures and are effortless to apply before they will accept them [38,39]. Naturally, people fear that AI may replace clinicians or take their jobs. This attitude might even guide students in their career choice [40,41]. Although this study found differences between groups where undergraduate male and SBC students were significantly more pessimistic, the overall score was quite neutral. Education and training in AI might further contribute to a differentiated view of the pros and cons of these technologies, including smart algorithms in medical applications [42].

This study, nevertheless, has some limitations. This study was conducted at a single institution although students from almost all medical faculties of German universities contributed to this investigation. Self-selection bias may exist due to voluntarily participation. Further, the quantitative survey consisted of about three-fourths women but only one-fourth men, indicating a gender bias corresponding to the gender distributions of students that reflects the situation in many medical faculties. The focus on the German educational system and the fact that only a small fraction of the total number of medical students filled out the online survey makes a generalization of the answers difficult. However, the thorough literature research, the extraction of relevant themes, and the number of interviews performed in the qualitative study phase were comparable to similar qualitative studies, including the number of items included in the survey, which was also accomplished with a similar level of substantiation [43]. The statements of the qualitative study part and questions of the quantitative study part may not always reflect clarity and comprehension. The reason is that those were entirely based on self-reported and subjective measures and therefore did not necessarily follow scientific semantics. The questionnaire for the quantitative study did not undergo a validated validation process. Instead, it underwent a face-validity process by the authors and was pretested in a pilot study, which has been used in other mixed-methods studies [18,36,43].

## 6. Conclusions

This study represents an important insight regarding digitization and AI-naive students and their perceptions, anxiety, and notions, which were solely based on personal interest, the participation of voluntary courses, or acquired from hidden curriculum. While the attitudes towards digitization in medicine were well-balanced between curricula groups, gender, and training stage, perceptions regarding AI were not. Although in comparison to other studies, AI illiteracy was lower, still, up to almost 40% of participants could not answer AI-related questions although differences in subgroups exist.

## 7. Implications

There is a broad understanding in the student cohort on the need to integrate education and training in digital applications and AI technologies in medicine. Therefore, it is recommended to integrate themes such as “digitization in medicine” as well as “AI” in the medical curriculum due to their increasing importance in health care. To cope with this aspect, the University Hospital Charité in Berlin started a project called “AI-Campus”, which offers courses on a voluntary basis and can be used by every member of medical faculty in Germany. Based on the results of our study, a more formal integration of AI and eHealth themes into health education would not only fit today’s requirements of cutting-edge patient care but would also suit medical students’ interests, as our study confirmed, and might reduce students’ digital illiteracy, which, however, has to be elucidated in another study.

## Figures and Tables

**Table 1:** Characteristics of the study cohorts.

Parameter	Specifics (Qualitative Study)	Specifics (Quantitative Study)
Students	*n* = 28	*n* = 1053
Age (years)	24.76 ± 3.05	23.7 ± 3.9
Gender distribution	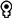 *n* = 17 (60.7%) 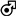 *n* = 11 (36.3%)	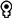 *n* = 779 (74.0%) 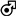 *n* = 274 (26.0%)
Semester		
(1–4) = Undergraduate	*n* = 10	*n* = 438 (41.6%)
(5–12) = Graduate	*n* = 18	*n* = 615 (58.4%)
Interview time (minutes/interviewee)	29.5 ± 2.6	25–30
PBC students	*n* = 13 (46.4%)	*n* = 490 (46.5%)
Gender	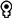 *n* = 5 (38.5%), 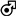 *n* = 8 (61.5%)	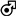 *n* = 153 (31.2%), 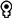 *n* = 337 (68.8%)
Age	26.9 ± 4.1	23.8 ± 4.0
SBC students	*n* = 15 (53.6%)	*n* = 563 (53.5%)
Gender	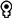 = 12 (80.0%), 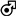 = 3 (20.0%)	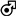 = 121 (21.5%), 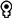 = 442 (78.5%)
Age	22.60 ± 2.03	23.7 ± 3.8

**Table 2:** Codes used in the qualitative study part.

Code	Descriptors	Subthemes
Health Apps	Professional health apps for medical decision finding Lay health apps giving diagnostic advise and therapeutic control	Doctor’s competitor, Doctor’s assistant Technical challenges Erroneousness (misleading) Patient’s assistant Information tool Patient–doctor alienation Economization of consultations
Wearables	Electronic devices to track physical metrics, consumer wearables	Effects on self-determination Medical device Motivation tool Self-controlling Health consciences Monitoring tool for physical fitness
Telemedicine	Telecommunication technology for remote health care	Simplification of doctor–patient interaction Monitoring tool 24/7 surveillance Amelioration of patient quality of life Enhances patient’s independence
Digitization in patient management	Electronic software solutions to aid the health care	Peer-to-peer communication Patient management Patient records Literature search Data management and transfer Digital literacy of users
Data protection	Safeguarding of important information	Data misuse Transparent patient Unnecessary restrain Patient health card
Robotics in medicine	Use of computerized or automated devices in health care	Doctor’s assistant Doctor’s competitor Support in diagnostic and analytic procedures Alienation of patients Legal responsibility
AI	Computer- or software-driven machines to perform activities normally thought to require intelligence	Doctor’s assistant Doctor’s competitor Support in diagnostic and analytic procedures Legal responsibility Distrust Lack of information

**Table 3:** Response (sum ± STD) from Likert scale responses to given questions. Statistical group comparison using the unpaired, two-tailed Wilcoxon rank-sum test.

Questions	PBC (*n* = 490)	SBC (*n* = 563)	Male (*n* = 274)	Female (*n* = 779)	Undergraduate (*n* = 438)	Graduate (*n* = 615)
Digitization makes doctors in diagnostic workup dispensable. 0 = do not know, 1 = false, 7 fully agreed.	2.50 ± 0.91	2.60 ± 1.00	2.72 ± 1.09	2.49 ± 0,91	2.54 ± 0.990	2.56 ± 0.94
group comparison	*p* = 0.096		*p* = 0.007 (alpha = 0.003)		*p* = 0.588	
Medical decisions can be digitally supported but must be finalized through the doctors because only they can fully assess the outcome. 0 = do not know, 1 = false, 7 fully agreed.	5.99 ± 1.29	5.91 ± 1.32	5.95 ± 1.43	6.00 ± 1.26	5.92 ± 1.45	6.02 ± 1.96
group comparison	*p* = 0.971		*p* = 0.940		*p* = 0.925	
Health apps and computer algorithms are for patients disturbing (0) or coherent (100).	50.1 ± 22.6	47.5 ± 22.4	51.4 ± 24.7	47.7 ± 21.6	45.4 ± 22.5	51.1 ± 22.2
Group comparison	*p* = 0.066		*p* = 0.018 (*)		*p* < 0.0001	
Health apps/computer algorithms are in medicine debilitating (0) or supportive (100).	63.4 ± 18.9	60.5 ± 18.6	66.0 ± 20.4	60.4 ± 17.9	60.9 ± 19.3	62.62 ± 18.33
group comparison	*p* = 0.003		*p* < 0.001		*p* = 0.325	
Digital self-diagnostics are for patients deleterious (0) or useful (100).	38.1 ± 23.4	35.2 ± 20.8	38.0 ± 22.5	36.0 ± 21.9	33.2 ± 21.7	39.0 ± 22.0
group comparison	*p* = 0.105		*p* = 0.244		*p* < 0.0001	
The multiplicity of health apps cause confusion. 0 = do not know, 1 = false, 7 fully agreed.	3.88 ± 1.96	4.25 ± 2.04	4.17 ± 1.96	4.02 ± 2.03	3.96 ± 2.02	4.16 ± 2.00
	*p* = 0.001		*p* = 0.449		*p* = 0.083	
Wearables can replace 24 h ECG and others in medical diagnostics. 0 = do not know, 1 = false, 7 fully agreed.	2.74 ± 1.35	2.70 ± 1.30	2.84 ± 1.29	2.68 ± 1.29	2.75 ± 1.39	2.70 ± 1,28
Group comparison	*p* = 0.540		*p* = 0.114		*p* = 0.753	

* = non-significant after Bonverroni correction of alpha error.

**Table 4:** Response (sum ± STD) from Likert scale responses to given questions. Statistical group comparison using the unpaired, two-tailed Wilcoxon rank-sum test.

Questions	PBC (*n* = 490)	SBC (*n* = 563)	Male (*n* = 274)	Female (*n* = 779)	Undergraduate (*n* = 438)	Graduate (*n* = 615)
Digital networks (including telemedicine) make face-to-face medical consultations unnecessary. 0 = do not know, 1 = false, 7 = fully agree.	2.40 ± 0.86	2.41 ± 0.84	2.57 ± 1.05	2.35 ± 0.76	2.43 ± 0.92	2.39 ± 0.80
Group comparison	*p* = 0.567		*p* = 0.006 (alpha = 0.003)		*p* = 0.925	
Would it be problematic for you as a doctor that you work more at the computer instead of directly interacting with the patient? 1 = yes, 2 = no, 3 = do not know.	1.42 ± 0.73	1.41 ± 0.74	1.36 ± 0.70	1.44 ± 0.75	1.41 ± 0.74	1.42 ± 0.74
Group comparison	*p* = 0.692		*p* = 0.116		*p* = 0.641	
What do you think: Does digitization in medicine reduce (0) or enhance (100) personal doctor–doctor communication?	49.8 ± 26.2	49.2 ± 25.7	52.1 ± 27.4	48.6 ± 25.3	48.8 ± 25.8	50.0 ± 26.0
Group comparison	*p* = 0.705		*p* = 0.076		*p* = 0.496	
What do you think: Do digital networks increase (0) or decrease (100) doctor–nurse communication?	40.5 ± 22.3	36.7 ± 20.7	40.3 ± 22.5	37.8 ± 21.2	37.5 ± 21.3	39.2 ± 21.8
Group comparison	*p* = 0.010 *		*p* = 0.213		*p* = 0.170	
How do you deal with a non-perfect health care system: Do you try learn the pitfalls in order to adapt yourself (0), or do you try to improve an imperfect system actively (100)?	58.8 ± 24.6	57.4 ± 23.9	55.1 ± 26.2	59.1 ± 23.5	60.6 ± 23.6	56.2 ± 24.5
Group comparison	*p* = 0.316		*p* = 0.068		*p* = 0.003	
Digitization in medicine lacks confidentiality and breaches private data security. 0 = do not know, 1 = false, 7 = fully agree.	3.08 ± 1.20	3.17 ± 1.26	3.07 ± 1.17	3.15 ± 1.25	3.22 ± 1.29	3.07 ± 1.18
Group comparison	*p* = 0.296		*p* = 0.155		*p* = 0.025 *	
Do you regard the statutory health card susceptible for fraud (0) or a tool to improve quality of patient-centered care (100)?	67.8 ± 20.1	66.7 ± 20.7	69.5 ± 21.1	66.4 ± 20.1	65.6 ± 19.9	68.4 ± 20.8
Group comparison	*p* = 0.467		*p* = 0.011 *		*p* = 0.007 *	

* = non-significant after Bonverroni correction of alpha error.

**Table 5:** (**A**–**C**) Response (% in numeric columns) to given questions. Statistical group comparison using the unpaired, two-tailed Wilcoxon rank-sum test (statistical calculation excluding column 1).

**A**									
**Comparator groups**	**Artificial Intelligence (AI) Leads to Loss of Medical Skills. AI is “Addictive”. 1 = Do Not Know, Range of Agreement: 2 = Rejection, up to 7 = Fully Agreement.**								
	**1**	**2**	**3**	**4**	**5**	**6**	**7**	**Mean ± STD**	***p***
PBC	13.3	16.7	22.4	16.1	20.0	8.2	3.3	3.50 ± 1.63	*p* = 0.033 * (alpha = 0.016)
SBC	9.9	18.5	21.1	16.2	16.5	23.5	4.3	3.68 ± 1.69	
male	8.8	16.4	23.4	17.2	19.7	11.3	3.3	3.70 ± 1.59	*p* = 0.226
female	12.5	18.1	21.2	15.8	17.6	10.9	4.0	3.57 ± 1.69	
undergraduate	13.9	18.9	21.5	13.9	18.7	9.4	3.7	3.47 ± 1.69	*p* = 0.033 *
graduate	9.8	16.7	22.0	17.7	17.7	12.2	3.9	3.69 ± 1.64	
**B**									
**Comparator groups**	**Particularly in diagnosing orphan diseases, AI outmatches physicians. 1 = Do Not Know, 2 = Range of Agreement: 2 = Rejection, up to 7 = Fully Agreement.**								
	**1**	**2**	**3**	**4**	**5**	**6**	**7**	**Mean ± STD**	***p***
PBC	31.8	10.2	15.7	12.0	14.3	12.2	3.7	3.18 ± 1.93	*p* < 0.0001
SBC	32.7	13.7	13.5	12.6	16.3	8.3	2.8	3.03 ± 1.85	
male	28.1	8.4	11.3	12.4	17.9	15.0	6.9	3.56 ± 2.05	*p* < 0.0001
female	33.8	13.4	15.7	12.3	14.5	8.5	1.9	2.94 ± 1.81	
undergraduate	38.4	14.6	12.8	12.8	11.2	7.3	3.0	2.78 ± 1.83	*p* < 0.0001
graduate	28.0	10.2	15.8	12.0	18.4	12.2	3.4	3.33 ± 1.90	
**C**									
**Comparator groups**	**AI will cause disaster rather than being useful. 1 = Do Not Know, Range of Agreement: 2 = Rejection, up to 7 = Full Agreement.**								
	**1**	**2**	**3**	**4**	**5**	**6**	**7**	**Mean ± STD**	***p***
PBC	19.0	25.9	30.6	14.5	5.3	3.5	1.2	2.77 ± 1.35	*p* = 0.158
SBC	17.8	26.1	25.4	17.2	7.5	4.1	2.0	2.91 ± 1.46	
male	6.6	38.0	29.6	12.0	7.7	3.6	2.6	2.97 ± 1.35	*p* = 0.002
female	22.5	21.8	27.2	17.3	6.0	3.9	1.3	2.79 ± 1.43	
undergraduate	21.5	21.2	24.4	17.4	8.4	4.3	2.7	2.94 ± 1.55	*p* < 0.001
graduate	16.1	29.4	30.2	15.0	5.0	3.4	0.8	2.88 ± 1.30	

* = non-significant after Bonverroni correction of alpha error.

## Supplementary Materials

The following supporting information can be downloaded at: https://www.mdpi.com/article/10.3390/healthcare10040723/s1, Figure S1: Students’ perceptions on “the internet-affine patient”; Table S1: Interview themes in 28 students of five German universities (qualitative study part); Table S2: Students’ perceptions on health apps (lay and professional health apps); Table S3: Students’ perceptions on wearables use by patients; Table S4: Students’ perceptions on telemedicine; Table S5: Students’ perceptions on digitization in patient management (hospital, ambulatory); Table S6: Students’ perceptions regarding data security; Table S7: Students’ perceptions regarding robotic and intelligence (AI) in medicine.
