# Supplementary material for: Medical Students’ Perceptions towards Digitization and Artificial Intelligence: A Mixed-Methods Study
Source: Healthcare (Basel). 2022 Apr 13;10(4):723. doi: 10.3390/healthcare10040723 (PMC9027704; doi:10.3390/healthcare10040723)

Supplementary Materials

Table S1 Interview themes in 28 students of five German universities (qualitative study part)

Reference group	Primary interview topic	Adjunct queries
Patients	Personal assessment of digitization from the patient perspective	Judgment of digitization in patient self-management including the interaction with the health-care system<br>Unities and pitfalls of digitization in medical self-diagnostics<br>Opportunities and risks of various digital tools in medicine, the patient might use:<br>• The internet affine patient<br>• Health apps<br>• Wearables
Physicians	Assessment of eHealth	Artificial intelligence<br>Digitization in treatment decission making<br>Digitization in the diagnostic workup<br>Aspects of eHealth:<br>• Electronic health records<br>• Patients health card<br>• Digitized archiving<br>• Digitization of administrative duties<br>Digitized communication (with peers, with patients, with health care providers etc.)<br>Telemedicine<br>Machines in health care
Students	Digitization as part of the medical curriculum	Lectures in eHealth<br>Introduction in digitization in medicine<br>Personal appreciation (technological progress vs nuisance) of eHealth

Table S2 Students perceptions on health apps (lay and professional health apps).

**Health apps: impact on the doctor's side (students' perspective)**
restrained:<br>• Professional health apps compete against doctor's knowledge and expertise<br>• Reservations towards digital evolution<br>• Being forced to constantly update medical knowledge is cumbersome<br>• Older doctors may refuse to use diagnostic apps<br>• Apps only supportive for technical enthusiasts<br><br>*undecided or positive*<br>• Apps are supportive for doctors' decision finding<br>• Apps are both: a challenge as well as chance<br>• Apps may be superior to doctors knowledge and expertise<br>• Apps as a useful quick-reference source<br>• Professional apps help to counterbalance lay health apps used by patients
**Health apps: impact on the patient's side (students' perspective)**
restrained<br>• Lay health apps as a competition to doctor's expertise<br>• Apps generate wrong diagnosis, reliability is questionable<br>• Apps alienate and perturb lay users, particularly when the doctor has a different view<br>• Apps may give wrong advise<br>• Apps may generate unnecessary doctor consultations and emergency visits<br>• Apps may prevent timely doctor consultations through wrong advise<br><br>*undecided or positive*<br>Health apps support the search of medical information<br>• Apps are both: a challenge as well as chance

Table S3 Students perceptions on wearables use by patients

**Wearables**
restrained<br>• Wearables weaken self-determination by influencing ones life schedule<br>• are not medical certified and may generate wrong signals<br>• may alienate and perturb users (inaccurate data even more so)<br>• To rely solely on wearables may cause an undesired dependency<br><br>*undecided or positive*<br>• perfect for physical self-assessment<br>• motivating to stay physically fit<br>• indicate patient's health consciences an helps ones self-determination regarding physical fitness<br>• Boon and bane<br>• help to improve patient therapeutic adherence<br>• may improve patient-doctor relationship if the use is consented<br>• support patients in self-controlling treatment success<br>• help patients to discern their physical limits<br>• some patients accept to be controlled by wearables but some not, both notions have to be tolerated by the doctor<br>• may help to detect medical problems early including emergency situation (epilepsy, arrhythmia)

Table S4 Students perceptions on telemedicine.

**Telemedicine**
restrained<br>• good examples (e.g. from Australia) not easily transferred to other countries<br>• not generally a good option in health care<br>• unclear liability issues<br>• lively patient-doctor contact generally better than virtual consultations<br><br>*undecided or positive*<br>• enable quick patient-doctor consultations and saves traveling time<br>• physical contact with health care institutions is less needed (saves resources)<br>• eases consultations with handicapped patients (with travel difficulties)<br>• Useful in sparsely populated states: easing patient-doctor consultations<br>• Helps paramedics in emergency situations through remote doctor consultation<br>• Supportive in remote medical monitoring of chronically ill<br>• Reduces physically consultations and ameliorate patient's quality of life<br>• Enhances patients' independence<br>• Eases number of doctors needed per population<br>• Enable 7/24 consultations<br>• Automation of data transfer and analysis saves working time<br>• Telemedicine, video-chats, symptom tracker support each other

Table S5 Students perceptions on digitization in patient management (hospital, ambulatory).

**Digitization in hospital and in out-patient care**
restrained
Prioritization of computer work causing negligence of patients
Personal resistance towards usages of digitized tools
Reduced personal communication with peers and nurse-doctor-interaction
Electronic patient management requires readiness from potential users
Digitization results in reduced personal nurse-doctor-interaction
Ubiquitous data access reduces the demand to memorize data
positive
Feed back on drug side effects, drug-drug-interaction, cheapest alternative
Electronic patient records: superiority over paper based records through e.g. flexible data access, reliable archiving and documentation, clarity, time saving
Easy lookup (patient data, med. literature) and high flexibility in usage
Easy communication with peers, between institutions and with patients (digitized consultation)
Software solutions supportive in diagnostics, therapy and documentation
Easy networking of different workflows, interactions of procedures

Table S6 Students' perceptions regarding data security.

**Data protection / security**
Concerns<br>You never know who uses personal data (e.g. health insurance companies) with grave consequences<br>General concerns of becoming a transparent citizen<br>Concern to loose owns health card with all the personal data<br>Concern of data loss of "critical health data" (e.g. HIV infection)<br>Data protection laws restrain doctor's work in quick patient handling<br>Concerns causes patients to become overcautious or secretive<br>Protection of personal (health) data is a general issue (WhatsApp, open WLAN) and needs to be addressed thoroughly<br>Unclear judicial responsibilities and possible legal consequences<br>Data collection and usage only with patients' consent and data access strictly regulated to avoid abuse<br><br>*No concerns*<br>No concerns, because patients need to open up to the doctor anyway<br>"Push this thought simply aside"<br>information, thus health data is only of small concern<br>Benefits of data collections outweigh concerns of data security

Table S7 Students' perceptions regarding robotic and intelligence (AI) in medicine

**Robotic in medicine**
Robots support surgeons (easing working conditions)<br>Diagnostic robots in direct patient application misses human interaction<br>Robots support doctors but will never replace them completely<br>Surgical robots allows remote surgical applications performed by distant experts<br>Surgical robots help to avoid human faultiness vs. are less fault-prone than year long experienced doctors<br>Surgical robots (with joystick operation) are fascinating<br>Robotic nurses support human nurses/doctors with simple tasks<br>Robots saves time for other more complicated tasks in patient care<br>Robots never compete with humans through lack of human interaction
**Artificial intelligence**
restrained<br>Unclear judicial responsibilities: doctor, hospital or software company ?<br>AI make doctors dependent and may result in loss of skills<br>AI lacks empathy and human touch<br>AI can get out of hand though autonomy (like in the movie iRobot)<br>Also AI has its limits particularly in rare and complex cases.<br>Intrinsic distrust against AI<br>AI lacks empathy and human touch<br>AI make doctors dependent and may result in loss of skills/knowledge<br>Computer and AI will never redundantize doctors ("absurd thought")<br>AI in direct patient contact may consternate elderly patients<br><br>*positive or undecided*<br>Great potential, support doctors decision finding. AI elevates doctors work on a new level<br>Doctors must be trained to understand underlying algorithms<br>Doctor must function as back-up when AI fails. Only doctors can make the ultimate medical decision not AI<br>boon and bane: ideal to support doctors but should not replace them<br>AI redundantize doctors, e.g. in radiology<br>Speeds up diagnostic work-up, frees doctor working time (e.g. Radiology)<br>AI is better than human doctors (e.g. in rare diseases), detect and avoid common human mistakes, is faster<br>Virtual reality and other AI applications are supportive and exciting<br>AI creates a different type of jobs in health care. AI helps to economize health care<br>AI can overtake many tasks, thus time in medical school could be trimmed<br>AI helps young inexperienced doctors<br>Never thought about AI, clueless (because not part of the curriculum)

Figure S1 Students' perceptions on "the internet affine patient"

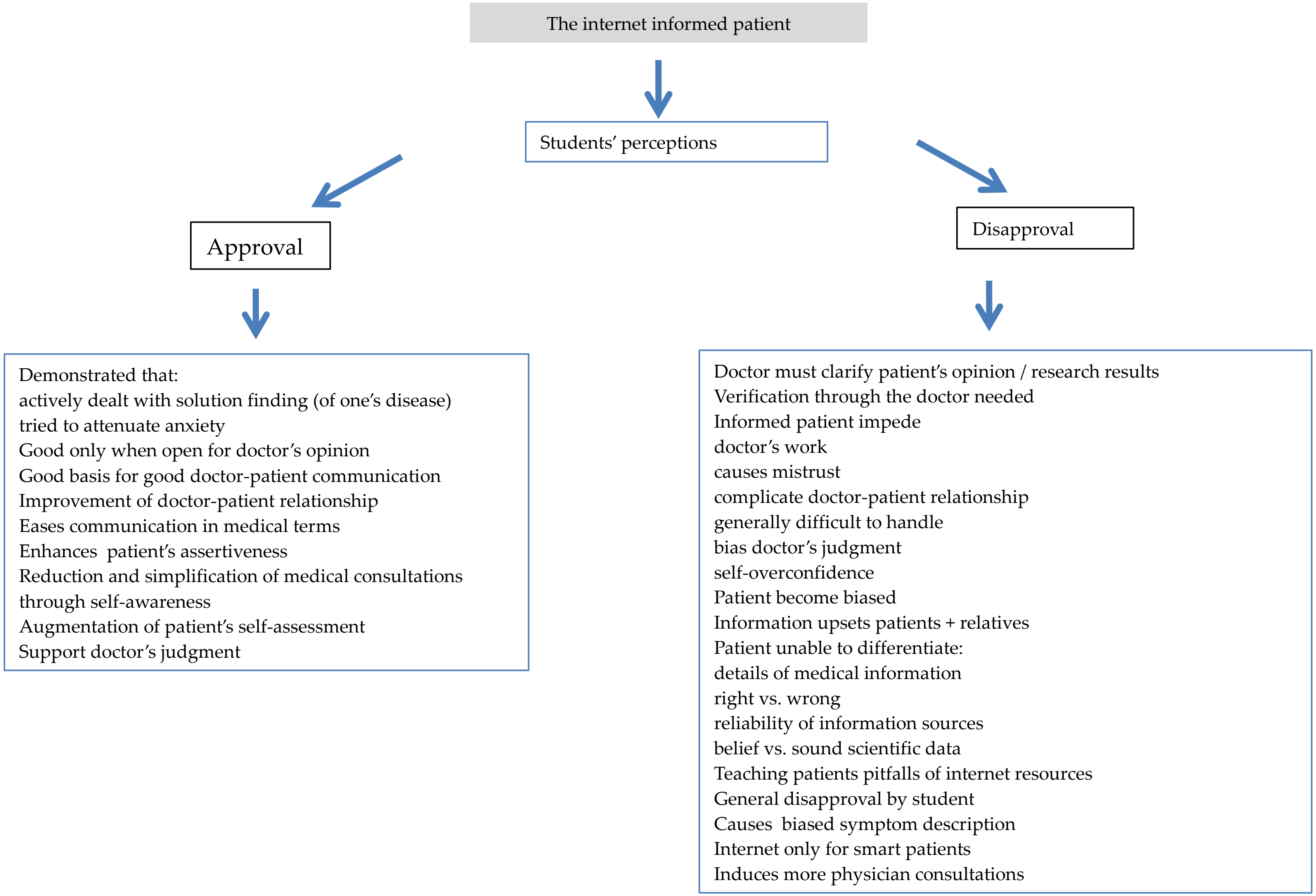

Supplement: Supplementary file 1 [file healthcare-10-00723-s001.zip › healthcare-1666105-supplementary.pdf]
